# Assessing Bowel Cleansing for Colonoscopy: Changing Our Focus to What Really Matters

**DOI:** 10.1155/2016/6754584

**Published:** 2016-05-09

**Authors:** Lawrence Hookey

**Affiliations:** Division of Gastroenterology, Queen's University, Kingston, ON, Canada K7L 5G2

Ever since colonoscopy was introduced, debate regarding how to best prepare the colon for the examination has been ongoing. We have evolved from the “Brown” prep, involving several days of purgatives (or have we? booster meds for days before colonoscopy are still being used and studied [1, 2]) to shorter and more palatable regimens. However, the continued interest in this field purely points to the lack of clear consensus or satisfaction with the current state. As I learned early in medicine, if there are 10 different therapeutic options, there is not yet a very good one, or all the others would have fallen away.

In the current issue, R. Mohamed et al. [3] report a randomized trial comparing split-dose polyethylene glycol solution (PEG) with more traditional dosing. This study was done well, with appropriate recruitment, sample size, randomization, patient follow-up, and outcomes, including bowel preparation measured through a validated scale [4]. The results confirm that split dosing is superior in cleansing to single-dosing PEG, with a large difference observed in Ottawa Bowel Preparation scores. However, tolerance of the preparations was still an issue, despite the authors' contention that they were “generally well tolerated.” Approximately 50% of each group reported nausea, with large proportions also reporting bloating and abdominal pain.

The authors' choice of split-dose regimen also deserves further discussion. In most of the current literature, “split-dose” refers to taking one-half of the preparation the evening before the procedure and one-half the morning of the procedure [5]. However, in the study by R. Mohamed et al. [3], for morning (before 10:00) colonoscopy patients, the doses were split over 8 h the day before, with the single-dose group taking their preparation at noon the day before. For cases performed after 10:00, the more traditional definition of split-dose was followed, with one dose at 20:00 the evening before and the second 5 h before the examination appointment. The preparation starting at noon the day before colonoscopy was doomed to have a poorer preparation because evidence clearly points to the fact that the closer the preparation is to the examination the better the preparation is [6, 7]. Interestingly, it is not this subgroup that drove the results, however, because the later cases were those who showed the greatest difference, again pointing to the necessity of having some preparation taken on the day of the procedure. Unfortunately, we are left with cleanliness and tolerance as the only reported outcomes, when in reality we want to know the effect on the patient (polyp and adenoma detection) and endoscopist (duration of endoscopy/time spent cleaning bowel or suctioning liquid).

The recent buzzwords in health care have been “patient-centred care,” with a shift to concentration on the patient experience and choices. Although the merits of this approach are, of course, true, it should also be done in conjunction with efficacy assessment. The drive to meet patient tolerance goals in colonoscopy has perhaps led us to focus too much on certain outcomes in colonoscopy preparation. While tolerance is key to acceptance of the procedure, we must start with efficacy. If we cannot safely assess and remove a significant majority of polyps present, then it is not worthwhile to have had a well-tolerated preparation and procedure. This argument, of course, can be made well beyond the preparation and include insertion techniques, completion rates, withdrawal times, and retroflexion. The best-tolerated colonoscopy is the one you do not need to undergo, and that would include the one that needs to be repeated due to poor preparation. Truly patient-centred outcomes must include polyp detection, repeat procedures, and shortened intervals of next surveillance cases, along with comfort scores such as the Nurse Assessed Colonoscopy (NAPCOMS). In the study by R. Mohamed et al. [3], patients scheduled earlier than 10:00 were not given the option of a split-dose on the presumption that this would be too taxing on them. However, other studies have not made this adjustment and have reported excellent results. We performed one such study [6] and found that patient education is perhaps the key to all of these processes, in which we tell patients “this is your one shot at screening/preventing colon cancer in a number of years, potentially your whole life – it is probably worth getting up at 4:30 to get it right.” Perhaps a future study should give patients information and options and record which regimen they choose.

Finally, how does the continued research investigating bowel preparation translate into the “real world”? Little to no evidence exists in this realm for colonoscopy, although in other fields when compared with the real world it almost always displays poorer results [8–10]—likely a combination of less patient education, broader patient group, and variation in instructions/prescribing behaviour. Development of patient education and reminder tools is necessary to try to offset these effects.

In conclusion, the study by R. Mohamed et al. [3] confirms that split dosing provides a better preparation. It should now be considered the standard of care for both high- and low-volume preparations. Future research needs to focus on patient-centred outcomes—broadly interpreted as polyp detection, tolerance, and patient preference, most likely in that order of priority.

*Lawrence Hookey*
